# The first international and the sixth national Iranian stroke congress event report

**Published:** 2014-07-04

**Authors:** Mehdi Farhoudi, Kaveh Mehrvar, Hadiseh Kavandi, Arash Aslanabadi

**Affiliations:** Neuroscience Research center, Tabriz University of Medical sciences, Tabriz, Iran

**Keywords:** International Congress, Stroke, tPA Therapy

The first and the sixth National Iranian Stroke Congress were held on 27-29 November 2013 in the Petrochemical Recreation Complex, Tabriz, Iran. The congress was supported by World Stroke Organization and Turkey Cerebrovascular Association and almost 100 physicians and researchers across Iran and other countries participated in the congress.

Dr. Afshin Divani from USA, Dr. Manouchehr Seyyedi Vafaei from Denmark, Prof. Olsen Niles from Denmark and Dr. Ozcan Ozdemir from Turkey were the international guests of the congress. The congress was managed by Prof. Mehdi Farhoudi as Chief and scientific secretary and Dr. Kaveh Mehrvar as executive secretary. This congress was supported nicely by the president of Iran Stroke Association Dr. Mohammad Reza Gheini.

The main goal of this congress was to gather stroke-related specialists and masters (neurologists, neurosurgeons, radiologists, internists, physical medicine specialists, general practitioners, etc.) as well as paramedic staff (nurses, speech therapists, physiotherapists, etc.) to contribute updating stroke-related practical knowledge with the latest scientific achievements and recent developments. The other aim of the congress was recognition of related problems and obtaining their solutions in prevention and treatment fields.

Totally, 220 abstracts were submitted to the congress, 30 selected for oral presentation and 171 for poster presentation. Furthermore, 25 invited lectures were also presented.

The milestone of this scientific meeting comprised four predominant sections.

**Oral Presentations**

The main focus of the congress was discussing the acute stroke management. In the mornings main lectures presented and other presentations planned afternoons. In this congress, domestic and foreign prominent masters and specialists pointed the main ideas about acute phase stoke management. New medical findings in the field of stroke management including diagnostic methods, the usual treatments of stroke and related protocols, importance of teamwork in stroke management were also discussed in detail.

**Panels**

In addition, besides the scientific works and presentations two panels were provided in the congress. The first panel was about “difficulties of thrombolytic therapy in Iran” and the second one discussed the “importance of the medical team working in acute stroke” to help stroke patients.

**Workshops**

During the 3-day congress, eight major workshops were held in various areas of acute stroke. The title of these workshops, include nursing care in stroke, intravenous thrombolytic therapy, intra-arterial thrombolytic therapy, how to check National Institutes of Health Stroke Scale, neurosonology, imaging in acute stroke, rehabilitation after acute stroke, and empirical models of stroke. The workshops were regarded with acceptable number of attending participants considering practical discussion of experts and specialists.

**Exhibition**

As a peripheral program of the congress, an exhibition of pharmacological companies active in the field of stroke treatment and management was held. In this exhibition, the pharmacological companies in both traditional and modern medicine, research companies in the field of dealing and management of stroke-related patients’ education presented their latest products.

Finally, this international annual congress not only provides new fields to discuss stroke-related causalities, treatments or management, but also attendance and participating foreign neurology masters and specialist contributes to establish an international relationship and connects Iran to science world and international neurology centers across the world.

## Conclusion

As the main goals of this congress, including updating target group information, discuss the problems and suggestion practical approaches through analyzing the oral presentations of the urban and foreign masters and ideas suggested as posters, the scheduled panels were investigated as above-mentioned.

Finally, as a consensus conclusion, the contents of the statement were listed as followings:

1. According to the increase of stroke incidence, considering more prevalence among young aged groups, and significant casualties thus to stroke, according to WHO warning, prophylactic assessments and related management should be considered through improving the point of view of all society and officials. As mentioned, the second cause of vascular disease mortality and the most prevalent fact of disability among adult age group is stroke. So that as a proposal, it is been expected to coin Aban 7^th^ coincides with October 29 - world stroke day - as the stroke day in the national calendar to improve society health and lower stroke prevalence by correcting the food habits, society culture, and lifestyles.

**Figure 1:**
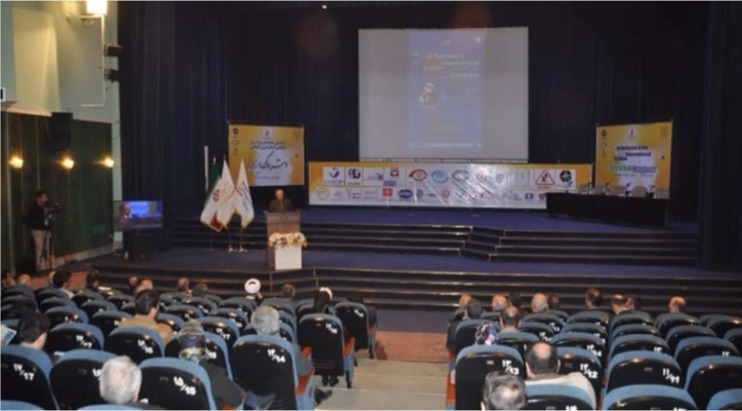
First day of congress and Dr. Shahram Dabiri’s lecture

1. According to either the proved effect of thrombolytic therapy in the acute phase of stroke in choice group patients and prominent role of this therapy in after stroke disability rehabilitation or considering the usefulness of the therapy due to its golden time, these points should be regarded:

- Stoke should be considered as one of the medical emergencies like trauma and myocardial infarction.
- Tissue plasminogen activator medication should be concluded in the conservative portfolio of health insurance.

**Figure 2:**
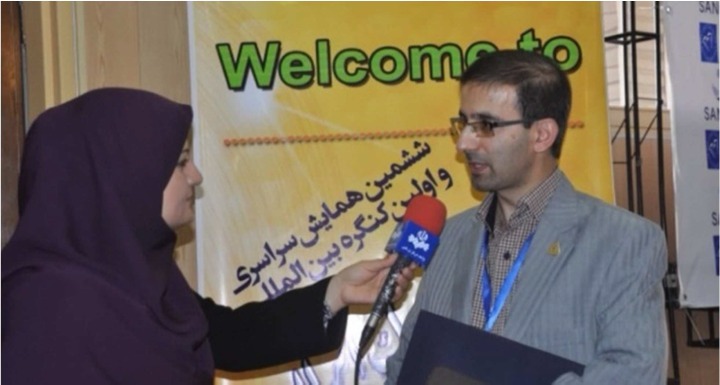
Scientific Secretary of the congress interview with Dr. Farhoudi, Central News Reporter

**Figure 3:**
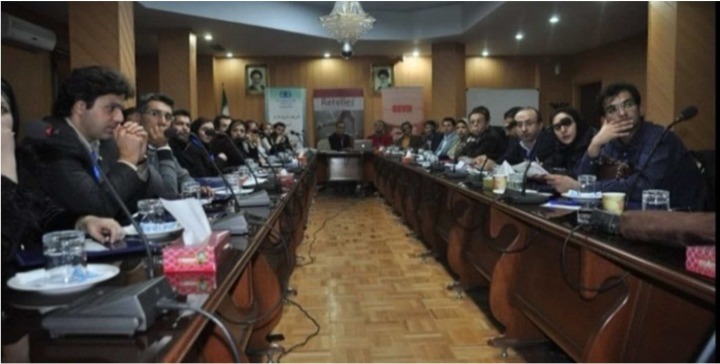
Side congress programs after lecture presentations

**Figure 4:**
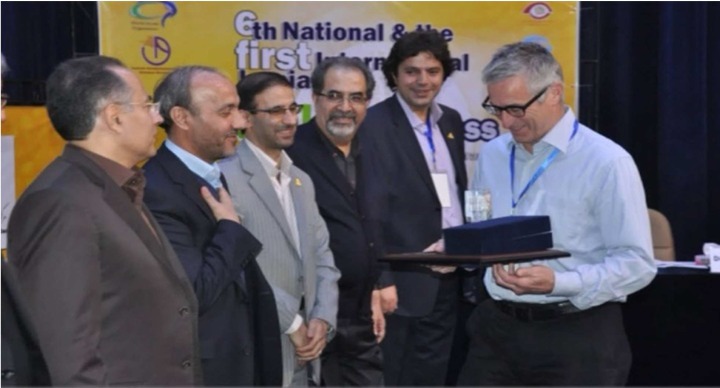
Appreciation ceremony and presenting gratitude certification plaque to premier posters and lectures

## Notes

**How to cite this article:** Farhoudi M, Mehrvar K, Kavandi H, Aslanabadi A. The first international and the sixth national Iranian stroke congress event report. Iran J Neurol 2014; 13(3): 193-4.

